# Developmental and reproductive toxicity assessment of sporoderm-removed *Ganoderma lucidum* spores

**DOI:** 10.3389/fcell.2025.1705415

**Published:** 2026-01-09

**Authors:** Junxiu Liu, Yisheng Song, Chuanhuai Chen, Jing Liu, Siming Zhang, Fang Liu, Ruiyu Tian, Jinjin Shao, Lili Zhang, Tingli Bian, Ruimin Sun, Li Yu, Shuizhen Pan, Yunxiang Chen, Yaoxian Xuan, Hanbo Wang, Zhenhao Li, Ying Chen, Lijiang Zhang

**Affiliations:** 1 Zhejiang Provincial Key Laboratory of Drug Discovery and Safety Evaluation for Inflammatory Chronic Diseases, Center of Safety Evaluation and Research, Hangzhou Medical College, Hangzhou, China; 2 Zhejiang Key Laboratory of High-level Biosafety and Biomedical Transformation, Hangzhou Medical College, Hangzhou, China; 3 Jinhua Shouxiangu Pharmaceutical Co., Ltd., Jinhua, China

**Keywords:** sporoderm-removed Ganoderma lucidum spores, reproductive toxicity, developmental toxicity, no-observed-adverse-effect level, medication safety

## Abstract

**Introduction:**

*Ganoderma lucidum* is a fungus used in traditional Chinese medicine with high medicinal value and is also widely used in modern healthcare. Its spores are reported to contain antitumor and anti-inflammatory properties, among other biological benefits; however, the thick spore wall limits its bioavailability. Sporoderm-removed *Ganoderma lucidum* spores (RGLS) offer improved bioavailability. However, data on their safety in pregnant and lactating populations remain limited, highlighting the need for developmental and reproductive toxicity (DART) assessment. We aimed to evaluate the developmental and reproductive safety of RGLS to support its clinical application in maternal and perinatal populations.

**Methods:**

Following ICH S5 (R3) guidelines, we conducted three non-clinical DART studies: embryo-fetal developmental (EFD) toxicity in rats, *in vitro* whole-embryo culture (WEC) in rabbits, and prenatal and postnatal toxicity (PPND) in rats. Female rats were administered RGLS (0.4, 1.2, and 4.0 g/kg/day) via oral gavage from gestation day (GD) 6 to GD17 (EFD) or to postnatal day (PND) 20. Rabbit embryos were cultured for 48 h in media containing 0.688, 0.963, and 1.238 mg/mL RGLS extract.

**Results:**

Our results showed no maternal toxicity, embryotoxicity, or teratogenicity in rats, apart from reversible drug-mixed feces. The offspring showed no adverse effects on growth, neurodevelopment (Morris water maze), or fertility. Rabbit embryos exhibited normal morphology and organ development. The no-observed-adverse-effect level of RGLS was 4.0 g/kg, which was approximately 20 times the intended clinical dose.

**Discussion:**

Overall, our study supports the safe use of RGLS in clinical applications for pregnant and lactating women, indicating that it can be added to a healthy diet.

## 1 Introduction

Traditional Chinese medicine, a remarkable legacy of China’s 5,000-year-old civilization, is widely acclaimed for its therapeutic efficacy and its vital role in safeguarding public health. *Ganoderma lucidum* stands out among other traditional Chinese medicines for its remarkable nutritional and medicinal value, with a documented use spanning over 2,000 years (Bishop et al., 2015). It is officially listed in both the Chinese Pharmacopoeia and the American Pharmacopoeia. With maturity, *Ganoderma lucidum*’s spores contain all the genetic information and have high medicinal and healthcare value (Ahmad et al., 2021b).

In recent years, the healthcare market has witnessed tremendous growth, and with increasing awareness around public health, the demand for *G. lucidum* spore powder has also surged. Numerous studies have demonstrated that the active components of *G. lucidum* spore powder exhibit a wide range of pharmacological effects, including, but not limited to, anti-inflammatory (Guo et al., 2021; Wen et al., 2022; Chen et al., 2023a), antioxidant (Zhong et al., 2022; Zhou et al., 2023), anti-tumor (Su et al., 2018; Cen et al., 2022; Shen et al., 2024), anti-depression (Zhao et al., 2021), liver protection (Jin et al., 2013; Chen et al., 2022), anti-atherosclerotic (Lai et al., 2020; Zheng et al., 2023), gut microbiota modulation (Leng et al., 2023), and improvement of cardiac insufficiency (Dai et al., 2019; Liu et al., 2021). These findings provide a solid scientific foundation for its use in the healthcare sector.

The biologically active constituents of *G. lucidum* spores (Xia et al., 2023), such as polysaccharides (Cai et al., 2017), triterpenoids (Wu et al., 2019), amino acids (Lian et al., 2023), nucleosides (Galappaththi et al., 2022), and fatty acids (Gao et al., 2012), are tightly enclosed within a rigid spore wall, which limits their bioavailability. Advanced sporoderm-removal technology facilitates the production of sporoderm-removed *G. lucidum* spores (RGLS). In our previous study, the chemical composition of RGLS was analyzed in terms of polysaccharides, total triterpenoids, and individual triterpenoid chromatographic fingerprinting, and the contents of polysaccharides and total triterpenoids were determined using the ultraviolet–visible spectroscopy (UV/VIS) method (Xia et al., 2023). This innovative processing method considerably enhanced the release and bioavailability of active compounds, thereby promoting better health outcomes (Li et al., 2020).

However, despite its broad pharmacological activity, toxicity research on *G. lucidum* spore powder remains limited. To address this gap, we conducted a 26-week potential reproductive toxicity study of RGLS in adult Sprague–Dawley (SD) rats. The results indicated that it had no significant toxic effects (Xia et al., 2023). Additionally, a randomized double-blind clinical trial involving 126 volunteers was conducted to evaluate the safety and effectiveness of the active ingredient β-glucan in *G. lucidum*, and the results showed no significant side effects (Chen et al., 2023b). These research findings provide strong evidence supporting the safety of *G. lucidum* spore powder. We comprehensively evaluated the effects of RGLS on embryonic-fetal and perinatal development in rats and rabbits and conducted three rigorously designed non-clinical developmental and reproductive toxicity (DART) studies to assess the adverse effects in both the F0 (parental) and F1 (offspring) generations.

## 2 Materials and methods

### 2.1 RGLS

The RGLS used in this study was provided by Jinhua Shouxiangu Pharmaceutical Co., Ltd. (batch number: 20200301). It was produced using advanced sporoderm-removal technology and primarily contained 21.4% crude polysaccharides and 5.7% total triterpenes.

### 2.2 Animals and maintenance

In accordance with the ICH S5 (R3) guidelines (ICH, 2020), we selected SPF-grade SD rats for the embryo-fetal developmental (EFD) and pre- and postnatal developmental (PPND) toxicity studies. We used rabbits as a second species for *in vitro* whole-embryo culture to supplement the EFD study. All experiments were conducted at the Safety Evaluation Research Center of Hangzhou Medical College.

SD rats were obtained from Zhejiang Weitong Lihua Laboratory Animal Technology Co., Ltd., and rabbits were sourced from the Xinjian Rabbit Farm in Juzhen, Dashi, and Xinchang counties. During the mating period, female rats were paired with male rats at a 1:1 ratio, with no more than two animals per cage. Pregnant and lactating female rats were housed individually.

All animals were maintained in a barrier facility under controlled environmental conditions: temperatures of 21.2 °C–24.1 °C, relative humidity of 41.0%–63.3%, a 12-h light/dark cycle, and at least 15 air changes per hour via central air conditioning. The facility was accredited by the Association for Assessment and Accreditation of Laboratory Animal Care (AAALAC, #001489). All procedures followed the Guide for the Care and Use of Laboratory Animals and were approved by the Institutional Animal Care and Use Committee (IACUC; approval numbers: GLP-2021-142 and GLP-2021-098).

### 2.3 Study and dose design

**FIGURE 1:**
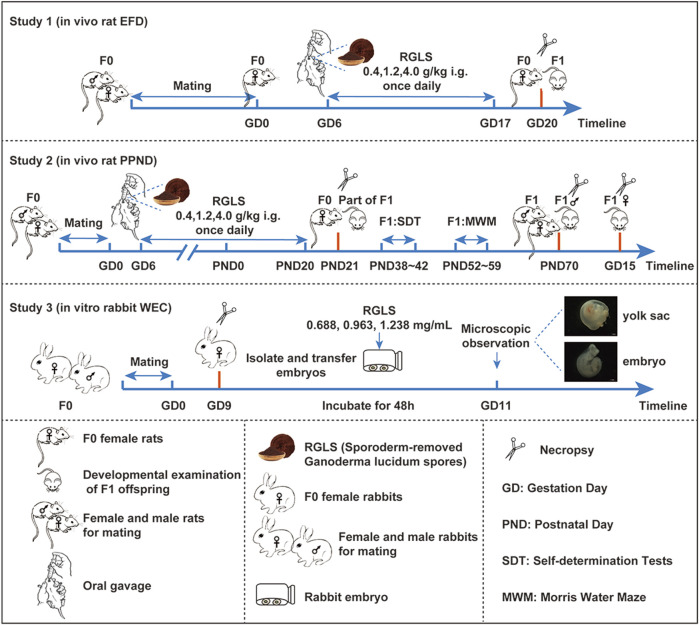
Study and dose design of reproductive toxicity tests.

We designed the study based on the ICH S5 (R3) guidelines and our prior experimental framework (Figure 1).

#### 2.3.1 Study 1: *in vivo* rat EFD

A total of 120 female and 80 male SD rats were mated. The doses administered to the rats were based on our previous 26-week repeated-dose RGLS toxicity study (Xia et al., 2023). Given the proposed clinical dose of 2 g/day per human adult, the equivalent dose for rats (using the Meeh–Rubner formula) is approximately 0.2 g/kg. Accordingly, the human equivalent doses for the low-, medium-, and high-dose RGLS groups were 2-, 6-, and 20-fold, respectively.

Ninety successfully mated female rats were randomly assigned to four groups: solvent control, low-dose (0.4 g/kg), medium-dose (1.2 g/kg), and high-dose (4.0 g/kg) RGLS groups, with 23, 22, 22, and 23 rats per group, respectively. Doses were administered once daily by oral gavage from gestational days 6–17 (GD6–GD17). On gestational day 20 (GD20), the F0 female rats were euthanized for embryo evaluation.

#### 2.3.2 Study 2: *in vivo* rat PPND

Another set of 120 female and 80 male SD rats were mated; 90 successfully mated female rats were randomly assigned to the same four groups as in study 1 (22, 23, 23, and 22 rats per group). Doses were administered once daily by oral gavage from GD6 through postnatal day 20 (PND20). On PND21, a gross examination was performed. One male and one female offspring from each litter were retained as the fertility assessment subgroup, while the remaining offspring underwent gross necropsy. The first generation (F1) rats in the fertility assessment subgroup underwent spontaneous locomotor activity testing (PND38–PND42) and Morris water maze testing (PND52–PND59). On PND70, fertility assessments were conducted, followed by mating. F1 male rats were necropsied post-mating, while female rats were necropsied on GD15 to assess embryonic development.

#### 2.3.3 Study 3: *in vitro* rabbit whole-embryo culture

Rabbit embryos at GD9 were cultured in medium supplemented with sodium chloride (control) or varying concentrations of RGLS extract (0.688, 0.963, and 1.238 mg/mL for low, medium, and high doses, respectively) for 48 h. Embryos showing yolk sac circulation and heartbeat were transferred to Hank’s solution for detailed morphological examination under a stereomicroscope.

The RGLS concentrations were determined from preliminary tests. The low-, medium-, and high-dose concentrations corresponded to 1.9-, 2.7-, and 3.5 times the maximum blood concentration (C_max_, based on the total ganoderic acid) observed with the 4.0 g/kg *in vivo* dose, equating to 38-, 54-, and 70 times the human equivalent dose, respectively.

### 2.4 Observation and measurements

#### 2.4.1 Study 1: rat EFD *in vivo*

The vaginas of female rats were observed daily after mating with male parental rats. The presence of a vaginal plug indicated successful mating. Throughout the study period, clinical signs, body weight, and food consumption were closely monitored in successfully mated pregnant F0 female rats. On GD20, the F0 female rats were euthanized and necropsied to assess the conditions of their tissues, organs, skin, and mucosa.

To determine the pregnancy status, the uterus and ovaries were isolated, and the uterus with the fetuses was weighed. The ovaries were collected, and the number of corpora lutea was recorded to determine the ovulation count and calculate pre-implantation loss.

Subsequently, we examined embryogenesis by dissecting the uterus to record the number of live, dead, and resorbed fetuses. The total number of implants was calculated accordingly. The gross appearance of the placenta was also observed, and the placental weight was recorded. After removal from the uterus, live fetuses were sexed, weighed, and measured for the crown–rump and tail lengths.

Approximately 50% of the live fetuses were used for visceral examination, and the remaining 50% were used for skeletal examination (only fetuses from the vehicle control and the high-dose group underwent these assessments). For visceral examination, fetuses were fixed in Bouin’s fluid, sectioned, and evaluated for developmental abnormalities and variations. For skeletal evaluation, fetuses were de-fleshed following the removal of visceral organs and interscapular fat, fixed in 95% ethanol, stained with Alcian Blue and Alizarin Red, and examined under a stereomicroscope. Abnormalities in the skull, sternum, vertebrae, ribs, limbs, pelvic girdle, and other skeletal structures were recorded. The fontanelle width, sagittal length, and ossification count were also measured.

#### 2.4.2 Study 2: rat PPND *in vivo*

Throughout the study, clinical signs, body weight, and food consumption of successfully mated F0 female rats were recorded. On PND21, gross anatomical examinations were performed on F0 female rats to assess potential abnormalities at the administration site, in visceral organs, or in the uterus.

For F1 generation rats, the clinical signs, survival rate, body weight, food consumption, and physiological and reflex development indicators were observed from birth (PND0) to weaning (PND21). During the physiological and reflex development examinations, we observed auricular separation starting from PND2, and the righting reflex was assessed. Negative geotaxis was evaluated beginning on PND6, along with incisor eruption from PND8 and hair outgrowth from PND9. The auditory startle reflex and air righting reflex were examined starting from PND11. Eye opening was observed from PND13, followed by the assessment of the pupillary reflex. Ear unfolding was monitored beginning on PND14. All developmental observations were recorded for each F1 rat until a positive result was achieved for each test.

At PND21, the F1 rats were divided into two groups: one for fertility assessment and the other for gross anatomical dissection. In the fertility subgroup, we evaluated the body weight, food consumption, sexual development, neurodevelopment, and fertility. From PND27, we observed the vaginal opening in female rats and preputial separation in male rats to assess sexual maturation of the F1-generation rats. After PND70, male and female rats from the fertility subgroup were mated. Following successful mating, male rats were euthanized for gross anatomical examination, while female rats were dissected on GD15 to examine uterine contents.

#### 2.4.3 Study 3: rabbit WEC *in vitro*

We humanely euthanized GD9 rabbits and extracted their serum and intact uteri after dissection. The excised uteri were transferred to sterile beakers filled with Hank’s solution. The uterine wall was carefully dissected at the orifice to expose the placenta, embryo, yolk sac, and uterine lining. Only healthy embryos with well-defined peripheral blood vessels, intact yolk sacs, and regular heartbeats were used for transplantation.

The selected embryos were placed into 100-mL culture bottles containing 7 mL of rabbit immediate centrifugation serum, 70 μL of double antibody solution, glucose at a concentration of 0.4 g/mL, and RGLS water-soluble extract at concentrations of 0.688, 0.963, or 1.238 mg/mL. The culture bottles were incubated in a temperature-controlled rotating incubator set at 38 °C and 25 rpm to facilitate continued embryonic development. A gas mixture of 20% O_2_, 5% CO_2_, and 75% N_2_ was introduced into the incubator before the start of culture and again after 2 h. Subsequently, a mixture of 5% CO_2_ and 95% O_2_ was introduced at 17, 29, and 41 h of culture, with each gas exchange lasting 2 min.

Embryo survival and malformations were assessed microscopically, and morphological scores were assigned for the yolk sac; brain; visual, olfactory, and auditory organs; branchial arch; maxillary and mandibular processes; heart; and limb buds following Carney’s embryo morphology scoring system (Carney et al., 2007). Additionally, we measured the crown–rump and head lengths of the embryos simultaneously as growth parameters.

### 2.5 Behavior tests

In this study, behavioral tests, including the spontaneous motor activity test and the Morris water maze test, were conducted on the F1-generation fertility subgroup to evaluate neurodevelopmental outcomes.

#### 2.5.1 Spontaneous motor activity test

This test was conducted between PND38 and PND42 using eight rats of each sex per group from the F1-generation fertility assessment subgroup. Each rat was individually placed in a test box and allowed to acclimate for 1 min before the 5-min observation period began. The following parameters were recorded and compared between groups: the activity duration in the open field and central area, time spent in the center, average speed, number of standing events, and grooming behaviors.

#### 2.5.2 Morris water maze test

The F1-generation fertility assessment subgroup rats were tested between PND52 and PND59, with eight rats of each sex per group. Using the Morris water maze apparatus and the TopScan animal behavior analysis system, we evaluated learning and memory by measuring latency to locate the platform during the 4-day training phase, along with the time to first cross the platform location and the total time spent in the target quadrant during the spatial probe trial.

### 2.6 Statistics and data analysis

Quantitative data, such as the body weight of F0-generation rats, weight gain during pregnancy, lactation, food consumption, gestation length, body weight and food consumption of F1 female rats, development milestones, spontaneous motor activity indicators, number of corpora lutea, implantation sites, live/dead/resorbed fetuses, mating duration, embryo count, and embryo morphology scores, were expressed as the mean ± standard deviation. One-way ANOVA was used to assess statistical significance.

If no significant overall group difference was found (*p > 0.05*), statistical analysis was concluded, although selected pairwise comparisons were still performed for certain indicators. When an overall difference was significant (*p < 0.05*), pairwise comparisons were conducted. Levene’s test was used to assess the homogeneity of variance. If the variance was homogeneous (*p > 0.05*), the LSD test was applied; if it was heterogeneous (*p < 0.05*), the Games–Howell test was used.

Categorical data, including the pregnancy rate, litter size, sex ratio, and mortality in the F0 generation, along with birth survival, PND4 survival, nursing survival, external malformation rate, mating rate, pregnancy rate, live birth rate, resorption rate, stillbirth rate, pre- and post-implantation loss rates, and modified Irwin’s behavioral grading in the F1 generation, were expressed as percentages. The chi-square test was used for significance testing. When significant overall differences were observed (*p < 0.05*), further pairwise comparisons were performed. Indicators with an incidence of 0 or 1 across all groups were not subjected to statistical analysis and were instead reported descriptively using fractions. The significance level (α) was set at 0.05.

## 3 Results

### 3.1 General toxicity assessment of RGLS in the F0-generation pregnant rats

In studies 1 (EFD) and 2 (PPND), drug-mixed feces were observed in the low-, medium-, and high-dose groups during RGLS administration in the F0-generation female rats. No other abnormal toxic reactions were noted. Drug-mixed feces, a common response to orally administered herbal medicines, resolved after RGLS discontinuation and was, therefore, attributed to its metabolic and physicochemical properties rather than to toxicity. No deaths were associated with RGLS administration in either study, except for one F0 female rat in the mid-dose group of study 2, which died from obstructed labor (autopsy revealed an enlarged placenta in the left uterine horn). Since obstructed labor is a spontaneous and episodic abnormality at birth in rats and occurred in only one case without a dose–response relationship, it was not attributed to RGLS.

**FIGURE 2:**
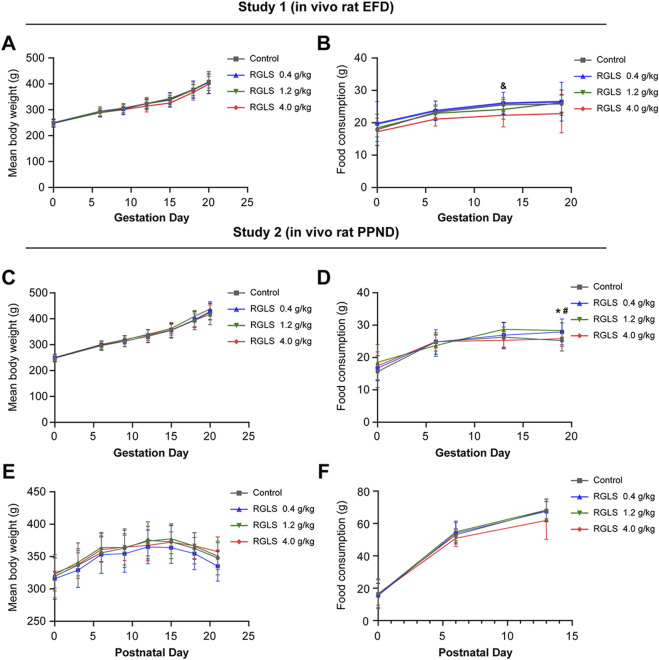
Average body weight and food intake of female rats of F0 in study 1 and 2. **(A)** The average body weight of F0 female mice during pregnancy in study 1 (*in vivo* rat EFD); **(B)** Food intake of female rats of F0 during pregnancy in study 1; **(C)** The average body weight of F0 female mice during pregnancy in study 2 (*in vivo* rat PPND); **(D)** Food intake of female rats of F0 during pregnancy in study 2; **(E)** The average postpartum body weight of F0 female mice in study 2; **(F)** Food intake of F0 female rats after delivery in study 2. Data were described by means ± standard deviation, and significance was determined by One-Way ANOVA. Compared with the solvent control group, RGLS in the low-dose group was *p < 0.05; compared with the solvent control group, RGLS in the medium-dose group was #*P < 0.05*; compared with the solvent control group, RGLS in the high-dose group was &*P < 0.05*.

In both studies, no significant toxic effects on the body weight or food intake were observed in F0 female rats at any RGLS dose (Figure 2). Across studies 1 and 2, body weights in the RGLS groups did not significantly differ from those in the solvent control group at any time-point (Figures 2A,C,E). For food intake, only a single time-point in study 1 showed reduced consumption in the high-dose group compared to that in controls (Figure 2B). In study 2, increased food intake was observed only on GD19 in the low- and medium-dose groups (Figures 2D,F), with no significant differences at other time-points. These minor variations in food intake were considered toxicologically insignificant due to their small magnitude and the lack of a dose–response relationship.

At the end, both studies showed that no significant abnormal changes were observed during gross examination of the tissues and organs of F0 female rats in any RGLS-administered dose group.

### 3.2 Evaluation of female fertility and embryo-fetal development toxicity of RGLS in rats

Study 1 (EFD) showed that RGLS had no significant toxic effects on female fertility or embryonic development. No significant differences were observed between the RGLS and control groups in the pregnancy rate, uterine and embryo weights, mean placental weight, number of corpora lutea, implantations, live fetuses, stillbirths, resorbed fetuses, or pre- and post-implantation loss rates (Table 1). Fetal measurements following cesarean section, fetal weight, body length, and tail length also showed no significant differences across groups.

**TABLE 1:** RGLS data on fertility and embryo formation in F0 female mice in rat EFD study.

Indicator	Control	RGLS 0.4 g/kg	RGLS 1.2 g/kg	RGLS 4.0 g/kg
Pregnancy rate	18/23 (78.3%)	18/22 (81.8%)	19/22 (86.4%)	16/23 (69.6%)
Weight of uterus and embryo (g)	77.58 ± 24.75	72.10 ± 28.05	80.27 ± 16.15	73.53 ± 25.98
Placental weight (g)	0.53 ± 0.08	0.55 ± 0.09	0.50 ± 0.05	0.53 ± 0.08
Corpus lutea	17.28 ± 2.67	16.06 ± 3.40	17.63 ± 2.97	17.63 ± 3.50
Implantation sites	14.50 ± 3.47	12.89 ± 4.81	14.95 ± 2.91	14.19 ± 4.42
Live fetuses	13.56 ± 4.33	12.00 ± 4.92	14.26 ± 2.79	13.44 ± 4.43
Live fetus rate (%)	91.8% ± 18.0%	91.4% ± 12.7%	95.4% ± 6.4%	93.7% ± 12.3%
Dead fetuses	0.00 ± 0.00	0.11 ± 0.47	0.00 ± 0.00	0.06 ± 0.25
Dead fetus rate (%)	0.0% ± 0.0%	2.8% ± 11.8%	0.0% ± 0.0%	0.4% ± 1.5%
Resorptions	0.94 ± 1.66	0.78 ± 1.06	0.68 ± 0.89	0.69 ± 0.95
Resorption rate (%)	8.2% ± 18.0%	5.9% ± 7.5%	4.6% ± 6.4%	6.0% ± 12.4%
Preimplantation loss	2.78 ± 2.02	3.17 ± 2.09	2.68 ± 2.50	3.44 ± 3.16
Preimplantation loss rate (%)	16.7% ± 14.0%	22.7% ± 21.9%	14.8% ± 13.0%	20.1% ± 20.6%
Post-implantation loss	0.94 ± 1.66	0.89 ± 1.08	0.68 ± 0.89	0.75 ± 0.93
Post-implantation loss rate (%)	8.2% ± 18.0%	8.6% ± 12.7%	4.6% ± 6.4%	6.3% ± 12.3%
Sex ratio (% male)	124/120 (103%)	114/102 (112%)	122/149 (82%)	101/114 (89%)
Fetal body weight (g)	3.57 ± 0.81	3.70 ± 0.95	3.54 ± 0.89	3.44 ± 0.91
Fetal body length (cm)	3.29 ± 0.33	3.32 ± 0.35	3.26 ± 0.32	3.25 ± 0.35
Fetal tail length (cm)	1.24 ± 0.08	1.25 ± 0.08	1.24 ± 0.08	1.22 ± 0.10

Pregnancy rate = pregnant female rats/female rats mated × 100%.

Live fetus rate (%) = live fetuses/implantation sites × 100%.

Dead fetus rate (%) = dead fetuses/implantation sites × 100%.

Resorption rate (%) = resorption/implantation sites × 100%.

Preimplantation loss rate (%) = (corpus lutea − implantation sites)/corpus lutea × 100%.

Post-implantation loss rate (%) = (implantation sites −live fetuses)/corpus lutea × 100%.

No external, soft tissue, or skeletal malformations were observed in fetuses from the control or high-dose groups. The incidence of all fetal abnormalities, including malformations, variations, and uncategorized abnormalities, did not differ significantly between the RGLS-treated and control groups (Table 2).

**TABLE 2:** Summary of fetal examination data in the rat EFD study.

Indicator	Control	RGLS 0.4 g/kg	RGLS 1.2 g/kg	RGLS 4.0 g/kg
External examination				
Fetuses/litters examined (n)	244/18	216/18	271/19	215/16
Total malformations	0/0	0/0	0/0	0/0
Total variations or uncategorized abnormalities	0/0	0/0	0/0	0/0
Soft tissue examinations				
Fetuses/litters examined (n)	117/18	/	/	102/16
Total malformations	0/0	/	/	0/0
Total variations or uncategorized abnormalities	2/2	/	/	3/2
Pyelectasis (bilateral) [V]	2/2	/	/	3/2
Skeletal examination				
Fetuses/litters examined (n)	127/18	/	/	113/16
Total malformations	0/0	/	/	0/0
Total variations or uncategorized abnormalities	115/18	/	/	109/16
Parietal, incomplete ossification [V]	10/5	/	/	8/4
Interparietal, incomplete ossification [V]	90/17	/	/	80/16
Occipital, incomplete ossification [V]	15/5	/	/	18/6
Metacarpal, incomplete ossification [V]	1/1	/	/	2/2
Metatarsal, incomplete ossification [V]	4/3	/	/	5/4
Sternum, incomplete ossification [V]	26/11	/	/	17/9
Sternum, unossified [U]	74/14	/	/	78/14
Short rib [U]	1/1	/	/	0/0
Multiple ribs [V]	1/1	/	/	5/4
Punctate rib [V]	1/1	/	/	5/4
Asymmetrical rib [V]	0/0	/	/	3/2
Pubis, unossified [U]	5/4	/	/	4/4
Pubis, incomplete ossification [V]	10/5	/	/	12/7
Ischium, unossified [U]	2/2	/	/	0/0
Ischium, incomplete ossification [V]	1/1	/	/	4/4
Thoracic vertebral centrum, dumbbell ossification [V]	73/16	/	/	70/16
Thoracic vertebral centrum, two-site ossification [V]	19/11	/	/	21/10
Thoracic vertebral centrum, unilateral ossification [U]	1/1	/	/	0/0
Lumbar centrum, dumbbell ossification [U]	1/1	/	/	0/0
Caudal centrum, unossified [U]	2/2	/	/	8/2
Sacral vertebra centrum, incomplete ossification [V]	1/1	/	/	0/0

Litters with the malformation rate (%) = litters with malformations/litters examined × 100%.

Litters with the variation rate (%) = litters with variations/litters examined × 100%.

Data are presented as the number of fetuses affected/number of litters affected. “/” indicates no corresponding observation.

[M] malformation, [V] variation, and [U] uncategorized abnormality.

These results suggest that RGLS does not exert toxic effects on female fertility or embryo-fetal development in rats and does not induce teratogenic effects.

### 3.3 General toxicity assessment of RGLS on prenatal and postnatal development in F0-generation rats (PPND study)

In study 2 (PPND), RGLS had no significant effect on the pregnancy rate of F0 female rats (Table 3), which is consistent with the findings of study 1 (EFD). Delivery data (Table 3) showed no significant differences among RGLS dose groups and the control group in delivery-related parameters, including the birth rate, dystocia rate, incomplete parturition rate, and gestational length. No abortions, preterm labor, or delayed deliveries were observed in any dose group, except for one F0 female rat in the medium-dose group that died during gestation due to obstructed labor. Since the incidence of such abnormalities was low and not dose-dependent, these events were considered unrelated to RGLS.

**TABLE 3:** Summary of maternal delivery and pup data in PPND study.

Indicator	Control	RGLS 0.4 g/kg	RGLS 1.2 g/kg	RGLS 4.0 g/kg
Pregnancy rate	18/22 (81.8%)	21/23 (91.3%)	22/23 (95.7%)	20/22 (90.9%)
Parturition rate	18/18 (100.0%)	21/21 (100.0%)	21/22 (95.5%)	19/20 (95.0%)
Dystocia rate	0/18 (0.0%)	0/21 (0.0%)	1/22 (4.5%)	0/20 (0.0%)
Incomplete yield	0/18 (0.0%)	0/21 (0.0%)	0/22 (0.0%)	1/20 (5.0%)
Gestation length (days)	21.6 ± 0.5	21.9 ± 0.4	21.5 ± 0.5	21.6 ± 0.6
Survival rate at birth	100.0%	98.7%	99.7%	99.6%
Survival rate on PND4	98.4%	99.3%	98.7%	98.2%
Survival rate in lactation (on PND21)	98.6%	100.0%	100.0%	98.6%
External malformation rate of pups born	0.00%	0.00%	0.00%	0.36%
Sex ratio of pups (♂/♀)	133/115	137/170	148/168	140/141
Pup body weight on PND0 (g)	6.65 ± 0.61	6.69 ± 0.63	6.74 ± 0.55	6.58 ± 0.71
Pup body weight on PND3 (g)	8.76 ± 1.17	8.97 ± 1.07	8.90 ± 1.24	8.33 ± 0.95
Pup body weight on PND6 (g)	14.63 ± 1.33	15.10 ± 1.54	15.16 ± 1.71	14.24 ± 1.28
Pup body weight on PND9 (g)	22.50 ± 1.60	23.28 ± 2.02	23.25 ± 2.20	22.22 ± 1.52
Pup body weight on PND12 (g)	31.05 ± 2.27	31.58 ± 2.49	32.18 ± 2.62	30.68 ± 1.86
Pup body weight on PND15 (g)	39.10 ± 3.17	39.79 ± 2.84	40.38 ± 2.97	38.61 ± 2.58
Pup body weight on PND18 (g)	46.78 ± 3.72	47.82 ± 3.63	48.64 ± 3.80	46.22 ± 3.47
Pup body weight on PND21 (g)	60.96 ± 4.67	61.29 ± 5.05	63.38 ± 5.26	58.88 ± 4.43

Pregnancy rate = pregnant female rats/female rats mated × 100%.

Dystocia rate (%) = dystocia female rats/pregnant female rats ×100%.

Incomplete yield (%) = incomplete female rats/pregnant female rats ×100%.

Appearance deformity rate (%) = number of appearance deformity pups/number of examined pups ×100%.

Survival rate at birth = number of pups born alive/number of pups born × 100%.

Survival rate on PND4 = number of live pups on PND4/number of pups born alive × 100%.

Survival rate in lactation = number of live pups on PND21/number of live pups after post-cull on PND4 × 100%.

Compare with blank control, one-way ANOVA *p < 0.05 and **p < 0.01.

In the F1 offspring, RGLS administration at all dose levels showed no significant effects on postnatal survival rates (at birth, at PND4, and during lactation) or on the sex ratio at birth (Table 3). One dead fetus in the high-dose group presented with exencephaly and tailless malformations. Since this was an isolated case with no obvious dose-related pattern, it was deemed a spontaneous malformation unrelated to RGLS exposure.

### 3.4 Evaluation of developmental toxicity of RGLS on F1-generation rats through PPND study

No significant developmental abnormalities were observed in physiological or reflex developmental indices in F1 rats, except for a slight delay in the onset of the auditory startle reflex in the high-dose group (Table 4), which occurred without any associated auditory abnormalities. Regarding the development of sexual characteristics in F1 rats, no differences were observed between groups in the age of vaginal opening in female rats. In male rats, the age of preputial separation was slightly later in the low- and high-dose groups than in the control group, with no significant difference between the medium-dose and control groups (Table 4). Given the lack of a clear dose–response relationship and the small magnitude of the changes, these variations were considered to have no toxicological relationship.

**TABLE 4:** Summary of postnatal development indices of F1 pups in the PPND study.

Indicator	Control	RGLS 0.4 g/kg	RGLS 1.2 g/kg	RGLS 4.0 g/kg
Total litters	18	21	21	18
Physical development				
Auricle separation	3.4 ± 0.6	3.3 ± 0.6	3.1 ± 0.7	3.4 ± 0.7
Incisor eruption	12.7 ± 1.2	13.3 ± 1.1	13.0 ± 1.1	13.4 ± 1.1
Appearance of Fur	12.7 ± 1.1	12.6 ± 1.0	12.5 ± 0.7	12.8 ± 0.8
Eyes opening	16.1 ± 0.6	15.7 ± 0.7	15.8 ± 0.7	16.2 ± 0.7
Pinna unfolding	17.1 ± 0.7	17.0 ± 0.7	17.1 ± 0.8	17.3 ± 0.5
Reflex development				
Plane correction	4.6 ± 1.4	4.0 ± 1.3	4.1 ± 1.3	4.9 ± 1.2
Negative geotaxis	8.8 ± 1.1	8.3 ± 1.1	8.5 ± 1.1	8.7 ± 1.1
Auditory startle	12.1 ± 0.6	12.1 ± 0.7	12.1 ± 0.7	13.1 ± 2.1*
Aerial righting	14.9 ± 1.7	14.3 ± 1.2	13.9 ± 1.3	14.2 ± 1.5
Pupillary reflex	16.1 ± 0.7	15.7 ± 0.8	15.8 ± 0.7	16.2 ± 0.7
Sexual development				
Vaginal opening	33.1 ± 2.1	33.0 ± 1.8	33.9 ± 1.7	33.4 ± 1.8
Preputial separation	39.9 ± 1.2	41.0 ± 1.5*	40.1 ± 1.4	41.7 ± 1.5**

Data are presented as the mean age (day at which 100% pups attained landmark) per litter ±standard deviation.

Compared with blank control, one-way ANOVA *p < 0.05 and **p < 0.01.

In terms of CNS development, none of the RGLS dose groups showed significant toxic effects in behavioral tests in F1 rats. In the autonomous activity test (Table 5), only the duration and path length in the center area were slightly reduced in the low- and medium-dose groups. Rats in the medium-dose group also spent less time standing in the box than the control group. Most other indicators showed no differences. These changes were considered individual fluctuations without any toxicological relationship.

**TABLE 5:** Autonomous activity detection of F1 pups in the PPND study.

Indicator	Control	RGLS 0.4 g/kg	RGLS 1.2 g/kg	RGLS 4.0 g/kg
Total fetuses	16	16	16	16
Total length of the route (mm)	7952.97 ± 3268.10	8124.77 ± 2208.62	6362.09 ± 2637.77	7136.81 ± 2058.97
Average speed in the box (mm/s)	26.61 ± 10.93	27.18 ± 7.39	21.29 ± 8.83	23.88 ± 6.89
Staying time in the central area (s)	12.44 ± 10.40	6.49 ± 5.17*	6.53 ± 6.92*	11.53 ± 7.49
Length of the route in the central area (mm)	566.76 ± 375.68	343.61 ± 246.54*	236.09 ± 209.37**	469.39 ± 320.76
Grooming (times/5 min)	2.81 ± 3.53	3.19 ± 2.14	3.44 ± 2.03	3.13 ± 2.45
Standing (times/5 min)	26.81 ± 10.78	26.38 ± 9.93	17.19 ± 5.75**	20.63 ± 7.68

Data are presented as the mean per individual ± standard deviation. Compared with blank control, one-way ANOVA *p < 0.05 and **p < 0.01.

**FIGURE 3:**
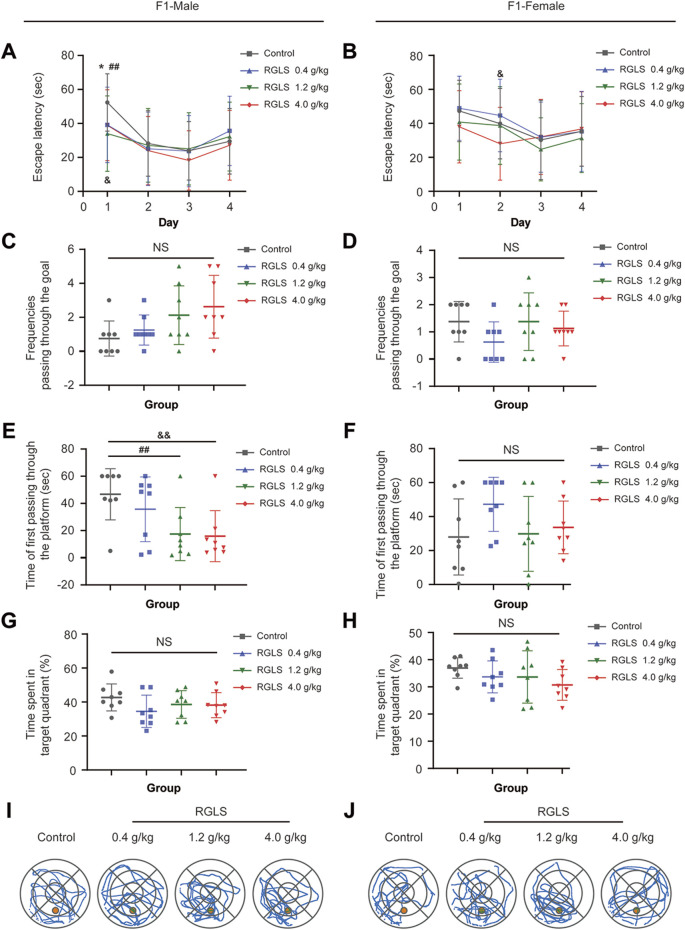
Investigation in the Morris Water Maze of F1 in in PPND study. **(A, B)** Correspond to the latency to locate the platform for male and female F1 rats during the four-day navigation trial within the Morris Water Maze; **(C, D)** Quantify the number of platform crossings by male and female F1 rats in the spatial probe trial on the fifth day of the Morris Water Maze; **(E, F)** Time of first passing through the platform by male and female F1 rats, respectively; **(G, H)** Time spent in target quadrant by male and female F1 rats, respectively; **(I, J)** The trace map of the 5th-day spatial exploration experiment by male and female F1 rats, respectively. Data were described by means ± standard deviation, and significance was determined by One-Way ANOVA. Compared with the solvent control group, RGLS in the low-dose group was * *P* < 0.05; compared with the solvent control group, RGLS in the medium-dose group was ##*P* < 0.01; compared with the solvent control group, RGLS in the high-dose group was &*P* < 0.05, && *P* < 0.01, compared with the solvent control group, NS indicating no significant difference.

In the Morris water maze test (Table 6; Figure 3), the latency of F1 male rats in all dose groups on the first day of training and that of F1 female rats in the high-dose group on the second day was shorter than that in the control group (Figures 3A,B). In the spatial exploration test, the frequencies passing through the goal did not differ significantly among all dose groups (Figures 3C,D). The time to the first crossing of the platform was shorter for F1 male rats in the medium- and high-dose groups than for the control group (Figure 3E), whereas no significant difference was observed in females (Figure 3F). Additionally, no significant differences were observed in the time spent in the target quadrant among the dose groups (Figures 3G,H). Both female and male rats administered RGLS showed an increase in the number of path crossings within the target quadrant compared to the control group (Figures 3I,J). These findings suggest that RGLS may enhance learning and memory in the offspring rats rather than produce toxic effects, which is consistent with results of a juvenile animal toxicology study conducted in our laboratory.

**TABLE 6:** Morris water maze behavioral detection of F1 rats in PPND study.

Indicator	Control	RGLS 0.4 g/kg	RGLS 1.2 g/kg	RGLS 4.0 g/kg
Latency of male rats				
Training rats	8	8	8	8
Total number of training (n)	32	32	32	32
Latency day 1(s)	52.39 ± 16.88	39.14 ± 22.20*	34.01 ± 22.20**	38.94 ± 20.87*
Latency day 2(s)	28.20 ± 19.31	25.12 ± 21.63	27.09 ± 21.70	24.00 ± 20.17
Latency day 3(s)	23.87 ± 17.25	23.67 ± 20.95	25.03 ± 21.28	18.22 ± 17.51
Latency day 4(s)	29.53 ± 19.28	35.65 ± 20.41	32.29 ± 20.25	27.16 ± 20.54
Space exploration of male rats				
Number of rats	8	8	8	8
Number of passes through the platform	0.75 ± 1.04	1.25 ± 0.89	2.13 ± 1.73	2.63 ± 1.85
Time taken for the first crossing of the platform (s)	46.71 ± 18.86	35.67 ± 23.82	17.43 ± 19.57**	15.90 ± 18.75**
Percentage of time in the target quadrant (%)	42.71 ± 7.94	34.53 ± 9.51	38.58 ± 8.15	38.15 ± 7.38
Latency of female rats				
Training rats	8	8	8	8
Total number of training (n)	32	32	32	32
Latency day 1 (s)	47.33 ± 18.11	48.87 ± 18.91	40.79 ± 22.44	37.95 ± 21.31
Latency day 2 (s)	39.81 ± 20.74	44.62 ± 21.37	38.68 ± 22.90	27.95 ± 21.40*
Latency day 3 (s)	30.25 ± 23.37	31.87 ± 20.67	24.69 ± 18.51	32.05 ± 22.05
Latency day 4 (s)	35.29 ± 20.56	35.20 ± 23.54	31.30 ± 20.30	36.68 ± 21.88
Space exploration of male rats				
Number of rats	8	8	8	8
Number of passes through the platform	1.38 ± 0.74	0.63 ± 0.74	1.38 ± 1.06	1.13 ± 0.64
The time taken for the first crossing of the platform (s)	27.96 ± 22.42	47.21 ± 15.88	29.82 ± 22.05	33.61 ± 15.48
Percentage of time in the target quadrant (%)	36.95 ± 3.75	33.69 ± 5.89	33.64 ± 9.63	30.74 ± 5.67

Data are presented as the mean per individual ±standard deviation. Compared with blank control, one-way ANOVA *p < 0.05 and **p < 0.01.

### 3.5 Fertility assessment of RGLS in F1-generation rats through PPND study

**FIGURE 4:**
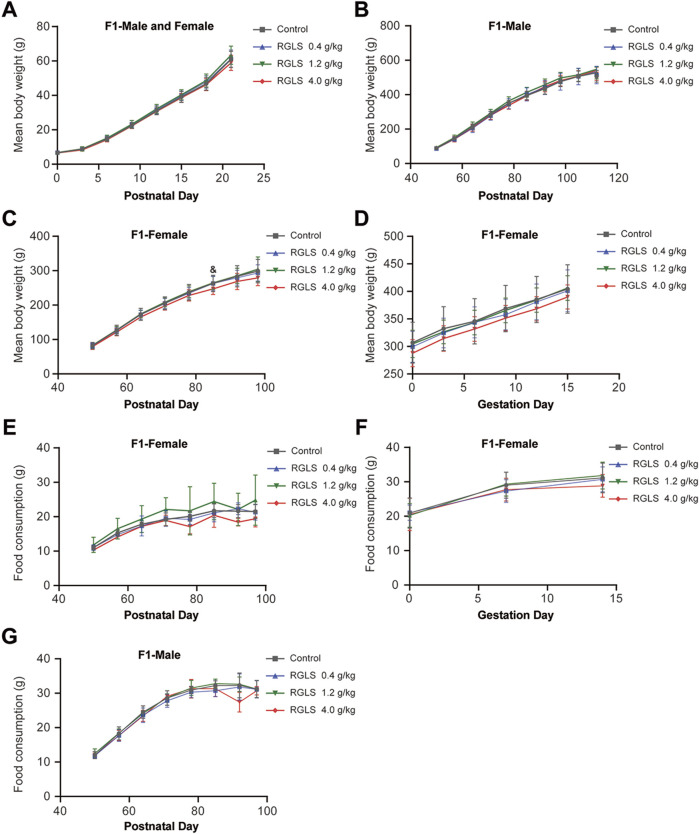
Body weight and food intake of F1 female rats in the PPND study. **(A)** Body weight of F1 rats after birth, including male and female rats. **(B)** Body weight of F1 male rats during the weaning period. **(C)** Body weight of F1 female mice during the weaning period. **(D)** Body weight of F1 female mice in gestation. **(E)** Food consumption of F1 female rats during the weaning period. **(F)** Food consumption of F1 female rats in gestation. **(G)** Food consumption of F1 male rats during the weaning period. Data were described as the means ± standard deviation, and significance was determined by one-way ANOVA. Compared with the control group, RGLS in the high-dose group was &*p < 0.05*.

No significant toxic effects were observed on the fertility indices, including mating, pregnancy, and embryo formation, in mature F1 male and female rats across all dose groups (Table 7). Body weights of F1 rats during lactation, weaning, and gestation did not differ significantly between the RGLS groups and the control group (Figures 4A–D); however, the body weights of F1 female rats in the high-dose group were slightly lower at PND 85 than those of the controls (Figure 4C). Since this occurred at only a single time-point and the fluctuation was minimal, it was considered toxicologically insignificant. Similarly, no significant differences were observed in food intake between F1 male and female rats in any dose group and those in the control group (Figures 4E–G).

**TABLE 7:** Summary of F1 fertility data in the PPND study.

Indicator	Control	RGLS 0.4 g/kg	RGLS 1.2 g/kg	RGLS 4.0 g/kg
Total pairs cohabited	18	21	21	18
Mated female rats	18	20	21	18
Pregnant female rats	18	18	20	18
Mating index (%)	18/18 (100.0%)	20/21 (95.2%)	21/21 (100.0%)	18/18 (100.0%)
Pregnancy (%)	18/18 (100.0%)	18/20 (90.0%)	20/21 (95.2%)	18/18 (100.0%)
Time to mating (days)	3.11 ± 1.64	2.35 ± 1.63	2.80 ± 1.51	2.65 ± 1.32
Corpus lutea	21.67 ± 4.91	20.44 ± 4.13	20.68 ± 4.20	20.12 ± 5.63
Implantation	15.39 ± 3.84	16.78 ± 2.65	16.95 ± 1.84	16.18 ± 3.13
Live fetuses	13.89 ± 4.56	15.83 ± 3.37	16.11 ± 1.91	15.41 ± 3.04
Live fetuses (%)	90.8% ± 19.0%	93.7% ± 8.5%	95.1% ± 6.9%	95.3% ± 4.7%
Dead fetuses	0.00 ± 0.00	0.00 ± 0.00	0.00 ± 0.00	0.00 ± 0.00
Dead fetuses (%)	0.0% ± 0.0%	0.0% ± 0.0%	0.0% ± 0.0%	0.0% ± 0.0%
Resorptions	1.50 ± 3.05	0.94 ± 1.21	0.84 ± 1.21	0.76 ± 0.75
Resorptions (%)	9.2% ± 19.0%	6.3% ± 8.5%	4.9% ± 6.9%	4.7% ± 4.7%
Preimplantation loss (%)	26.5% ± 21.4%	16.5% ± 11.9%	15.7% ± 15.1%	16.1% ± 18.4%
Post-implantation loss (%)	9.2% ± 19.0%	6.3% ± 8.5%	4.9% ± 6.9%	4.7% ± 4.7%

Mating index = female rats mated/female rats cohabited × 100%.

Pregnancy (%) = pregnant female rats/female rats mated × 100%.

Live fetuses (%) = live fetuses/implantation sites × 100%.

Dead fetuses (%) = dead fetuses/implantation sites × 100%.

Resorptions (%) = resorption/implantation sites × 100%.

Preimplantation loss (%) = (Corpus lutea − implantation sites)/corpus lutea ×100%.

Post-implantation loss (%) = (implantation sites − live fetuses)/corpus lutea × 100%.

Data in each group are presented as the mean per litter ±standard deviation, *p < 0.05 and **p < 0.01.

### 3.6 Evaluation of the toxicity of RGLS on the development of GD9 rabbit embryos *in vitro*

In accordance with the ICH S5 (R3) guidelines and to further support the findings of the EFD toxicity study, we carried out an *in vitro* assay using a second species, the rabbit (study 3). We carefully isolated GD9 rabbit embryos and incubated them for 48 h in rabbit immediate centrifugation serum containing different concentrations of water-soluble RGLS extract (0.688, 0.963, and 1.238 mg/mL). We evaluated the rabbit embryos under a microscope using Carney’s embryo morphology scoring system.

**FIGURE 5:**
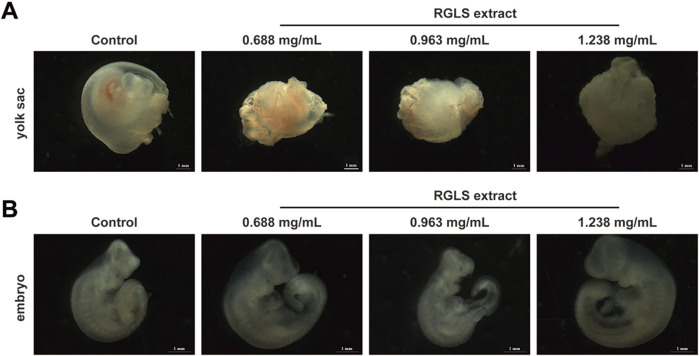
Morphology of the yolk sac and embryo of rabbit WEC on GD11 *in vitro.* **(A,B)** Morphology of yolk sac and embryo under microscope in the control group and RGLS extract in each dose group (0.688, 0.963, and 1.238 mg/mL), respectively.

The results showed normal development in embryos from all RGLS dose groups, with no malformations or dysplastic changes compared to the control group. Morphological parameters did not differ significantly between the RGLS and control groups, except for a slightly longer crown–rump length in the 0.688 mg/mL dose group (Table 8). Microscopic examination revealed that embryos in all groups were almost completely encased by yolk sacs, with visible capillary networks and robust blood circulation (Figure 5A). The embryos displayed complete body curvature, a fully closed neural tube, and three to four gill arches, indicating normal morphological and organ development (Figure 5B). These findings indicate that RGLS extracts at 0.688, 0.963, and 1.238 mg/mL did not induce developmental toxicity or teratogenic effects in rabbit embryos *in vitro*.

**TABLE 8:** Rabbit WEC morphological scores of RGLS on GD11.

Indicators	Control	RGLS 0.688 mg/mL	RGLS 0.963 mg/mL	RGLS 1.238 mg/mL
Embryo number	8	8	8	8
Head length (mm)	1.57 ± 0.17	1.74 ± 0.20	1.64 ± 0.17	1.67 ± 0.26
Crown arm length (mm)	4.20 ± 0.44	4.69 ± 0.26*	4.44 ± 0.32	4.44 ± 0.39
Somite number	30.75 ± 1.58	31.25 ± 1.28	29.00 ± 1.41	29.88 ± 1.13
Yolk-sac circulation	1.00 ± 0.00	1.00 ± 0.00	1.00 ± 0.00	1.00 ± 0.00
Yolk-sac blood vessels	4.13 ± 0.35	3.88 ± 0.64	3.75 ± 0.71	3.50 ± 0.53
Closed yolk sac	3.13 ± 1.133	3.38 ± 0.74	3.00 ± 0.00	3.38 ± 0.52
Buckling	3.88 ± 0.83	4.38 ± 0.52	3.38 ± 1.06	3.88 ± 0.83
Forebrain	3.75 ± 0.46	4.00 ± 0.00	3.63 ± 0.52	3.88 ± 0.35
Mesencephalon	4.00 ± 0.93	4.88 ± 0.35	3.88 ± 0.64	4.00 ± 0.76
Tritencephalon	4.50 ± 0.76	4.50 ± 0.53	3.75 ± 0.71	3.75 ± 0.71
Visual organ	3.75 ± 0.46	4.00 ± 0.53	3.50 ± 0.53	3.75 ± 0.46
Olfactory organ	1.00 ± 0.00	1.00 ± 0.00	1.00 ± 0.00	1.00 ± 0.00
Auditory organ	4.25 ± 0.89	4.00 ± 0.00	3.38 ± 0.52	3.63 ± 0.52
Branchial arch	4.13 ± 0.35	3.88 ± 0.35	4.00 ± 0.00	4.00 ± 0.00
Maxillary process	4.00 ± 0.00	4.00 ± 0.00	3.88 ± 0.35	4.00 ± 0.00
Mandibular process	3.75 ± 0.46	4.00 ± 0.00	3.50 ± 0.53	3.75 ± 0.46
Heart	3.50 ± 0.53	3.50 ± 0.53	3.13 ± 0.35	3.38 ± 0.52
Limb bud	3.75 ± 0.46	3.75 ± 0.46	3.13 ± 0.35	3.25 ± 0.46
Total head score	20.63 ± 1.19	21.38 ± 0.52	18.72 ± 2.12	19.38 ± 1.60
Total score	53.13 ± 5.14	54.13 ± 2.10	48.25 ± 3.45	50.25 ± 2.66

Compared with blank control, one-way ANOVA *p < 0.05.

## 4 Discussion

*Ganoderma lucidum*, a traditional Chinese medicinal herb with a long history and notable therapeutic value, transmits all its genetic characteristics through its spores. These spores, though minute (4 μm–6 μm), are encased in a tough outer wall composed primarily of chitin and polysaccharides. This structure significantly hinders the human body’s ability to digest and absorb the spore’s nutrients when consumed directly. In recent years, advances in spore wall-removal technology have greatly enhanced the release and bioavailability of the spore’s active components, thereby maximizing their pharmacological efficacy (Kou et al., 2023; Lu et al., 2020; Zhang et al., 2020).

Substantial progress has been made in pharmacological research on *G. lucidum* spore powder. For instance, one study demonstrated that it can precisely target the ferroptosis pathway, effectively inhibiting the development of oral squamous cell carcinoma (Wu et al., 2023). Another study showed that *G. lucidum* spore powder significantly reduces atherosclerosis and aortic calcification in mice by optimizing cholesterol excretion and inhibiting RUX2-mediated osteogenesis (Zheng et al., 2023). Additionally, it can modulate the gut microbiota, suppress the TLR4/Myd88/NF-κB inflammatory pathway, and effectively combat obesity and hyperlipidemia in mice fed a high-fat diet (Sang et al., 2021). Notably, during the COVID-19 pandemic, *G. lucidum* spore powder gained attention for its antiviral properties (Ahmad et al., 2021a), its ability to improve myocardial injury (Kuok et al., 2013), and its protective effects against cardiac dysfunction (Liu et al., 2021), offering hope to patients suffering from COVID-19-related myocarditis (Patone et al., 2022).

With growing research into the pharmacological effects of *G. lucidum* spores, public interest and demand for its related products have increased, making safety evaluations critically important. In previous studies, our team evaluated the long-term toxicity of RGLS and found that repeated administration over 26 weeks caused no toxic or adverse effects in adult SD rats. These findings offer important guidance for patients with chronic conditions who may require prolonged RGLS treatment (Xia et al., 2023).

However, data on the safety of RGLS use during pregnancy and lactation remain limited. According to the March of Dimes Foundation, approximately eight million children are born each year with serious genetic defects, accounting for 6% of live births globally, of whom three million die before the age of five (Walani and Biermann, 2017). Given that medication during pregnancy is a key factor that can contribute to fetal malformations, rigorous safety evaluation is essential to prevent tragedies similar to the thalidomide incident (Ito et al., 2010; Ren et al., 2021).

To address this gap and in accordance with the ICH S5 (R3) guidance, a non-clinical reproductive and developmental toxicity assessment was conducted in Sprague–Dawley rats to characterize the potential effects of RGLS on human reproduction and development. This DART study strengthens the safety-evaluation framework for RGLS and provides foundational evidence to inform its clinical use in pregnant and lactating women.

In recent years, increasing attention has been directed toward the effects of environmental pollutants and industrial chemicals on reproductive health. Phthalates, organophosphates (Gao et al., 2025), and nanoplastics (Lin et al., 2023) present in atmospheric particulate matter have been implicated in reproductive impairment through the disruption of sex-hormone regulation and endocrine signaling. In addition, the surfactant perfluorooctanoic acid (Zhou et al., 2022) has been associated with mitochondrial dysfunction, which may in turn compromise early embryonic development and oocyte quality in the offspring. Within this broader context, systematic evaluation of the reproductive and developmental toxicity of new therapeutics remains essential. In this study, the non-clinical safety of RGLS during pregnancy and lactation was comprehensively assessed using a multi-tiered experimental framework, including EFD toxicity studies, PPND studies, and rabbit embryo *in vitro* culture.

In the EFD toxicity study, pregnant rats received 0.4, 1.2, or 4.0 g/kg RGLS daily by oral gavage. No overt toxicological reactions were observed in the treated dams, and reproductive performance remained unaffected. Transient, drug-related alterations in fecal consistency occurred during the dosing period but resolved rapidly after cessation; this response is likely attributable to the metabolic handling and physicochemical properties of RGLS rather than to intrinsic toxicity.

Across all the administered dose levels, RGLS elicited no adverse effects on embryonic growth, external morphology, or visceral and skeletal development, and no teratogenic findings were detected. These outcomes contrast with the developmental toxicities reported for multiple environmentally derived contaminants. Based on these results, the NOAEL for maternal and embryo-fetal toxicity was identified as 4.0 g/kg, which is approximately 20-fold higher than the human equivalent dose. Collectively, these findings provide foundational preclinical evidence that can inform the reproductive-risk assessment of RGLS during pregnancy.

According to ICH S5 (R3), negative EFD findings from a single species should be supplemented by data from a second species, including data generated using validated alternative *in vitro* methods. Theunissen et al. (2016) reported that embryo-fetal lethality is more prevalent in rabbit models, whereas growth retardation, fetal variations, and malformations occur more frequently in rat models. A two-species EFD design, therefore, reduces the likelihood of false-negative outcomes arising from species-specific sensitivities and increases the overall predictive value. To fulfill this requirement, a rabbit WEC assay, an established *in vitro* alternative model for developmental toxicity, was performed. At RGLS extract concentrations of 0.688, 0.963, and 1.238 mg/mL, rabbit embryos exhibited normal morphological progression with no significant differences in developmental scores relative to the controls. These findings align with the absence of developmental toxicity observed in the *in vivo* rat EFD study, collectively strengthening the evidence that RGLS does not elicit developmental toxicity.

From GD6 to PND21, F0 rats received daily administration of RGLS at the same dose levels. Consistent with the EFD study, only transient changes in fecal consistency were noted in F0 dams, with no additional signs of toxicity. In the F1 generation, RGLS exposure did not adversely influence the developmental parameters, including reflex acquisition, survival, sex ratio, external morphology, body weight, food consumption, physiological and behavioral development, sexual maturation, or fertility. A slight delay in the auditory startle response was observed in a small number of animals at the highest dose; however, this effect normalized over time and was not considered toxicologically relevant. Necropsy of F0 and F1 animals revealed no RGLS-related pathological alterations. In the Morris water maze test, RGLS-exposed F1 offspring demonstrated a trend toward improved learning performance, suggesting a potential positive influence of RGLS on neurodevelopment.

Overall, the NOAEL for reproductive and developmental toxicity in F1 rats was identified as 4.0 g/kg, which is approximately 20-fold higher than the human equivalent dose. These findings provide substantial preclinical evidence that informs the perinatal safety profile of RGLS. Nonetheless, several limitations should be acknowledged. Interspecies physiological and metabolic differences may restrict direct extrapolation to humans. In addition, although the study characterized morphological and basic physiological endpoints of reproductive development, limited insight was gained into the underlying molecular pathways. Potential long-term consequences of RGLS exposure in offspring, including metabolic outcomes later in life, also remain unexamined. Future studies should investigate the mechanisms contributing to the absence of observable reproductive and developmental toxicity and assess whether RGLS confers protective effects under defined stress conditions. For early clinical studies, reference to FDA and EMA guidance, application of an appropriate safety factor to the NOAEL, and determination of a conservative maximum recommended starting dose will be essential. Development of human-relevant *in vitro* platforms, such as organoid or pluripotent stem cell-derived systems, could further refine the safety evaluation of RGLS in a human physiological context.

Although the molecular mechanisms and long-term effects of RGLS require continued investigation, current evidence supports a favorable reproductive and developmental safety profile. These observations indicate that RGLS may represent a promising therapeutic option for appropriate patient populations without apparent reproductive or developmental risk. In the context of increasing concern regarding environmental reproductive toxicants, the favorable safety characteristics of RGLS are particularly notable. Advancement to clinical development is, therefore, reasonable, provided that regulatory guidance is rigorously followed.

## 5 Conclusion

In this study, we comprehensively evaluated the reproductive and developmental toxicity of RGLS across a range of doses in SD rats, generating important data to support its safety profile in pregnant and lactating populations. The results indicated no significant toxicological effect. The results from our non-clinical study provide substantial evidence supporting the safety of RGLS during pregnancy and lactation. The non-clinical DART findings offer a strong scientific foundation for subsequent clinical evaluation and enhance our understanding of RGLS safety in maternal and perinatal populations.

## Data Availability

The original contributions presented in the study are included in the article/supplementary material; further inquiries can be directed to the corresponding authors.
